# Genistein cooperates with the histone deacetylase inhibitor vorinostat to induce cell death in prostate cancer cells

**DOI:** 10.1186/1471-2407-12-145

**Published:** 2012-04-11

**Authors:** Cornel J Phillip, Christopher K Giardina, Birdal Bilir, David J Cutler, Yu-Heng Lai, Omer Kucuk, Carlos S Moreno

**Affiliations:** 1 Department of Pathology and Laboratory Medicine, Emory University School of Medicine, Atlanta, GA, USA; 2 Graduate Program in Genetics and Molecular Biology, Emory University, Atlanta, GA, USA; 3 Winship Cancer Institute, Emory University, Atlanta, GA, USA; 4 Department of Hematology and Medical Oncology, Emory University School of Medicine, Atlanta, GA, USA; 5 Department of Human Genetics, Emory University, Atlanta, GA, USA

**Keywords:** Prostate cancer, Soy, Natural compounds, Epigenetics, Apoptosis

## Abstract

****Background**:**

Among American men, prostate cancer is the most common, non-cutaneous malignancy that accounted for an estimated 241,000 new cases and 34,000 deaths in 2011. Previous studies have suggested that Wnt pathway inhibitory genes are silenced by CpG hypermethylation, and other studies have suggested that genistein can demethylate hypermethylated DNA. Genistein is a soy isoflavone with diverse effects on cellular proliferation, survival, and gene expression that suggest it could be a potential therapeutic agent for prostate cancer. We undertook the present study to investigate the effects of genistein on the epigenome of prostate cancer cells and to discover novel combination approaches of other compounds with genistein that might be of translational utility. Here, we have investigated the effects of genistein on several prostate cancer cell lines, including the ARCaP-E/ARCaP-M model of the epithelial to mesenchymal transition (EMT), to analyze effects on their epigenetic state. In addition, we investigated the effects of combined treatment of genistein with the histone deacetylase inhibitor vorinostat on survival in prostate cancer cells.

****Methods**:**

Using whole genome expression profiling and whole genome methylation profiling, we have determined the genome-wide differences in genetic and epigenetic responses to genistein in prostate cancer cells before and after undergoing the EMT. Also, cells were treated with genistein, vorinostat, and combination treatment, where cell death and cell proliferation was determined.

****Results**:**

Contrary to earlier reports, genistein did not have an effect on CpG methylation at 20 μM, but it did affect histone H3K9 acetylation and induced increased expression of histone acetyltransferase 1 (HAT1). In addition, genistein also had differential effects on survival and cooperated with the histone deacteylase inhibitor vorinostat to induce cell death and inhibit proliferation.

****Conclusion**:**

Our results suggest that there are a number of pathways that are affected with genistein and vorinostat treatment such as Wnt, TNF, G2/M DNA damage checkpoint, and androgen signaling pathways. In addition, genistein cooperates with vorinostat to induce cell death in prostate cancer cell lines with a greater effect on early stage prostate cancer.

## Background

Among American men, prostate cancer is one of the most prevalent malignancies, accounting for 241,000 new cases and 34,000 deaths in 2010 [1], underscoring the importance of exploring new therapeutic approaches, targets, and the fundamental biological processes of the progression of this disease. Genistein is the major component (50 %) of soy isoflavones found in soybeans with 40 % daidzein and 5–10 % glycitein [2]. Genistein has been studied extensively and shown to have exciting antitumor activities including inhibition of tyrosine kinases, angiogenesis and proliferation [3-6], telomerase activity [7], oncogene function [8], and non-specific inflammation pathways [8,9], as well as induction of apoptosis [8,10]. The anticancer activity of genistein *in vivo* and *in vitro* has been demonstrated for carcinomas of the prostate [5,11-13], oral cavity [14], skin [15], bladder [6], and vulva [16]. Evidence suggests clearly that genistein has pleitropic effects on cancer cells, but the critical mechanisms of action remain ill defined.

Recent studies have suggested that gene promoter CpG methylation can be prevented or reversed by soy isoflavones [17]. *Fang et al.* reported that genistein (2–20 μM) inhibited cell growth, reversed DNA hypermethylation, and reactivated RARβ, p16^INK4a^, and MGMT in prostate cancer LNCaP and PC3 cells [17]. Genistein (20–50 μM) also dose-dependently inhibited DNA methyltransferase activity, showing substrate- and methyl donor-dependent inhibition. In addition, other studies have indicated that genistein can reactivate silenced genes such as the BTG3 tumor suppressor via CpG demethylation and increased H3K9 histone acetylation [18-20]. These results indicate that genistein and related soy isoflavones can reactivate epigenetically silenced genes, suggesting an additional mechanism for their therapeutic effects in cancer.

Genistein is attractive as a demethylating agent and as a potential therapeutic agent compared to the nucleoside analogue 5-aza-2’-deoxycytidine (5-aza) due to its minimal toxicity. 5-aza has been shown to have some effectiveness in treating various cancer types, but side effects such as neutropenia and myelosuppression are sometimes observed in patients [21]. Genistein is a naturally occurring compound that is well tolerated with no known toxicities [22,23]. Previous studies have shown that genistein can sensitize prostate cancer cells to treatment with the chemotherapeutic drug docetaxel [8]. Despite many studies regarding genistein, its effects on demethylation and impacts on gene expression are not completely understood.

The Wnt and Notch pathways are often deregulated in prostate cancer and are important in the progression of this disease. Several negative regulators of the Wnt pathway including the adenomatous polyposis coli (APC), secreted frizzled-related protein *(*SFRP1), dickkopf-related protein 3 (DKK3), and sex determining region Y-box 7 (SOX7) are hypermethylated in a high proportion of prostate cancers [24-26]. In addition, another negative regulator of Wnt that reduces tumor growth, cell migration and invasion, Wnt inhibitory factor 1 (WIF1), has also been suggested to be hypermethylated in prostate cancers [27].

In this study, we tested the hypothesis that demethylation and induction of Wnt inhibitory genes by treatment with genistein might result in decreased activity of the Wnt signaling pathway. We also tested whether genistein might cooperate with other compounds such as the histone deacetylase (HDAC) inhibitor vorinostat [28] to induce apoptosis. Surprisingly, we found that contrary to earlier studies, genistein has no effect on CpG methylation at physiologically relevant concentrations using methylation specific PCR and whole genome methylation analysis. Nevertheless, we did observe that genistein affected histone acetylation and cooperates with vorinostat to induce apoptosis even better than combined treatment of vorinostat with 5-aza. Furthermore, whole genome methylation analysis and whole gene expression analysis of the effects of genistein, vorinostat, or the combination of both compounds on gene expression in ARCAP-E (epithelial) and ARCAP-M (mesenchymal) prostate cancer cells provided insights into the mechanisms of action of genistein in cells prior to and after undergoing the epithelial-to-mesenchymal transition (EMT) [29].

## Methods

### **Cell lines and prostate patient samples**

DU145, PC3, and LNCaP were obtained from the American Type Culture Collection (Manassas, VA). DU145, PC3, and LNCaP cells were maintained in T-media (Gibco) supplemented with 10 % FBS, 200 mM L-glutamine, and pen-strep antibiotics. ARCaP-E and ARCaP-M cells were purchased from Novicure Biotechnology and propagated in MCaP-Medium (Novicure Biotechnology, Inc, Birmingham, AL), supplemented with 5 % FBS, 200 mM L-glutamine, and pen-strep. Cells were counted on a hemocytometer after staining with Trypan Blue. Additionally, prostate patient samples from primary tumors were obtained under IRB approved protocols from Emory University Hospital. Clinical characteristics of these samples are provided in Supplemental Table S1 (Additional file 1: Table S1.)

## Materials

Genistein and 5-aza-2’-deoxycytidine (5-aza) were obtained from Sigma Aldrich (St. Louis, MO), ICG-001 was obtained as a gift from the University of Southern California in the laboratory of Dr. Michael Kahn, University of Southern California, N-[N-(3,5-Difluorophenacetyl)-L-alanyl]-(S)-phenylglcine t-butyl ester (DAPT) was obtained from Santa Cruz Biotechnology, Inc (Santa Cruz, California), and N-Hydroxy-N’-phenyloctanediamide (vorinostat) was obtained from Toronto Research Chemicals, Inc (North York, ON, Canada). Each drug was dissolved in dimethylsulfoxide (DMSO) and stored in aliquots at −20°C.

### **Methylation specific PCR (MSP)**

DNA was extracted using the DNeasy Blood and Tissue Kit (Qiagen, Valencia, CA). Bisulfite treatment was performed using the EZ DNA Methylation Direct kit (Zymo Research Corp, Orange, CA) and MSP was performed using the EZ DNA Methylation startup kit (Zymo Research Corp, Orange, CA). Methylated and unmethylated primers were designed and optimized for APC, DKK3 SOX7, WIF1, SFRP1, and SFRP2 (Additional File 1: Table S2). In addition, the size of the product and the annealing temperature for each primer pair are indicated.

### **Chromatin immunoprecipitation (ChIP) assay**

ChIP assays were performed as described previously [30] using 90 % confluent ARCaP-E cells, fixed in 1 % formaldehyde and sonicated for 10 minutes. Sonicated chromatin was immunoprecipitated with H3 acetyl K9 antibody (Abcam, San Francisco, CA,USA) and rabbit IgG (Vector Laboratories, Burlingame, CA, USA) and collected with protein G agarose beads (Upstate, Temecula, CA, USA). Cells were washed twice with IP Dilution Buffer, TSE-500, LiCl Detergent, and TE buffer in listed order. Beads were then eluted and PCR was performed on the purified DNA using the primers in Additional File 1: Table S3.

### **Cell death assay**

After indicated drug treatments, cells were trypsinized and washed twice with 1X PBS. Cells were subsequently resuspended in 1X Annexin V binding buffer and incubated with Annexin V-FITC (Pharmingen Biosciences, San Diego, CA, USA) for 15 minutes at room temperature. 400 μl of Annexin binding buffer and 1 μg/mL propidium iodide (PI) were then added. The total cell death was measured using the BD FACSCalibur system (BD Biosciences Pharmingen, San Diego, CA, USA).

### **Quantitative real time RT-PCR**

Total RNA was isolated from cells using the RNeasy Mini Kit (Qiagen, Valencia, CA). After RNA isolation, total RNA was reverse transcribed to cDNA and quantitative real-time PCR was performed using the iQ SYBER Green Supermix on a Bio-Rad iCycler in a 25 μl total volume reaction. Primer sequences for Real-time RT-PCR are listed in Additional file 1: Table S4. The relative gene expression levels were determined by the ∆∆C_T_ method comparing threshold cycles with β-actin or 18 S as a normalization control.

### **Bisulfite sequencing**

Samples were sequenced on an Ion Torrent Personal Genome Machine at the Cancer Genomics Shared Resource at the Winship Cancer Institute. Sample DNAs (600 ng), which had been previously used for the whole genome methylation analysis, were bisulfite modified using the EZ DNA Methylation-Direct kit (Zymo Research) as before. Methylation-specific primers were designed using MethPrimer software [31] to amplify a 175-bp region of the WIF1 promoter (Forward: AATAGTTTTGGTTGAGGGAGTTGTA, Reverse: ACCAACAAACACAAAAAAATACTCC). PCR reaction was performed using 1 μl of bisulfite-treated DNA templates, 1 X Zymo Taq PreMix (Zymo Research), and 0.4 μM of each primer in a total volume of 25 μl. Cycling parameters for PCR were 95°C 5 min, followed by 40 cycles of 95°C 30 s, 58.2°C 30 s, and 72°C 30 s, followed by the extension step at 72°C 10 min, and 4°C hold. Amplicons were gel-extracted using the Qiagen Gel Purification kit (Qiagen), and submitted to the Emory Winship Cancer Institute Cancer Genomics Shared Resource for Ion Torrent sequencing, which was performed as described [32]. Sequencing data was analyzed using PEMapper software (Cutler D, Patel V, Mondal K, Ramachandran D, Steinberg K, Shetty A, Zwick M: PEMapper: A New Approach to Identifying Genetic Variation in Second-Generation Sequencing Studies, submitted)*.*

### **Whole genome methylation profiling**

DNA was isolated from three independent experiments in which ARCaP-E and ARCaP-M cells were treated with DMSO control, 20 μM genistein, or 1 μM 5-aza for six days. Fresh drugs and media were changed every other day. Additionally, PREC cells (Lonza, Basel, Switzerland) were used as a normal human prostate cell line control. Total DNA was submitted to the Emory Winship Cancer Institute Cancer Genomics Shared Resource for analysis with Illumina 27 K CpG Methylation Arrays that interrogate 27,578 CpG loci > 14,000 genes using the Illumina Beadstation 500 instrument. After data normalization, Genome Studio software (Illumina) was used to compute β values (range 0–1) defined as β = methylated signal/(methylated + unmethylated signal). Probes were filtered to include only those with changes in β values > 0.2, and significance analysis of microarrays (SAM) software [33] was used to determine statistically significant changes in methylation. SAM analysis was completed with two-class unpaired settings, 500 permutations, and FDR <1 %.

### **Whole genome expression profiling**

RNA was isolated from three independent experiments in which ARCaP-E and ARCaP-M were treated with DMSO control, 20 μM genistein for 6 days, vorinostat for 48 hrs, or a combination of vorinostat (48 hrs) plus genistein (6 days). Total RNA was submitted to the Emory Winship Cancer Institute Cancer Genomics Shared Resource for processing and hybridization to Illumina Human HT-12 v3 Expression BeadChips that interrogate 48,804 probes. After hybridization and data normalization, significance analysis of microarrays (SAM) software was used to determine significant differences in gene expression. SAM settings were two class unpaired, 500 permutations, and a minimum fold change of 1.5 at FDR <1 %.

### **Immunoblotting**

DMSO and genistein treated cells were collected and centrifuged at 1000 rpm for 5 minutes, and the pellets were washed twice with 1X PBS. Cells were lysed using lysis buffer. Lysis buffer consisted of 0.137 mol/L NaCl, 0.02 mol/L TRIS (pH 8.0), 10 % glycerol, 1 % NP40, and a protease inhibitor cocktail obtained from Promega (San Luis Obispo, CA, USA). A mix of cell lysates (50 μg) and laemmli sample buffer (Bio Rad, Hercules, CA) were made and then heated at 99°C for 3 minutes. Proteins were separated by SDS-PAGE electrophoresis and transferred to nitrocellulose for immunoblotting. After transfer, nitrocellulose membrane was blocked for 1 hr at room temperature using blocking buffer from Li-Cor Biosciences (Lincoln, NE, USA). Membrane was incubated with primary antibody Histone acetyltransferases 1 (HAT1) (Santa Cruz, Santa Cruz, CA) overnight at 4°C and then washed three times with 1X PBS and 0.1 % Tween. To control for equal loading, GAPDH (Cell Signaling, Danvers, MA) was used as a loading control. Then the nitrocellulose membrane was incubated with a secondary fluorescent antibody (LI-COR Biosciences, Lincoln, NE, USA). Using the manufacturer’s protocol, membranes were imaged and quantitated using the Odyssey imaging system (LI-COR Biosciences, Lincoln, NE, USA).

## Results

### **Wnt inhibitory genes are methylated in prostate cancer patient samples**

**Figure 1:**
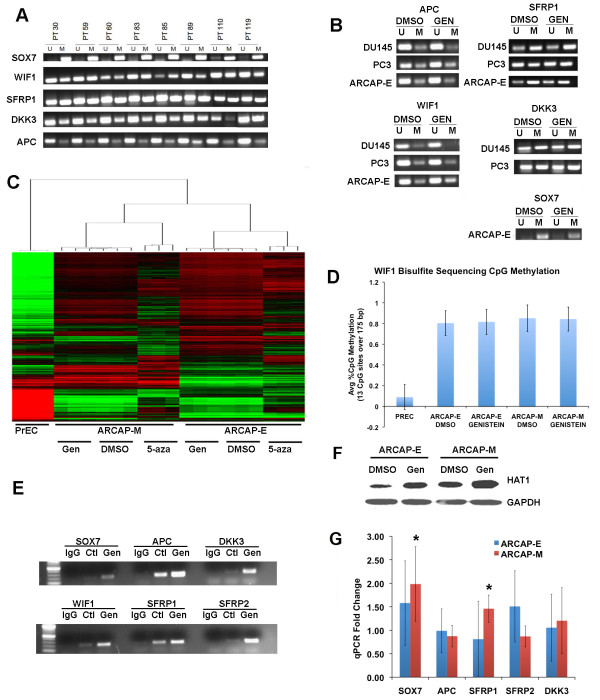
**Genistein treatment induces hypermethylated Wnt-inhibitory genes via H3K9 acetylation, not CpG demethylation. (A)** Wnt Inhibitory genes are methylated in prostate cancer patient samples. MSP of Wnt inhibitory genes was performed on genomic DNA derived from paraffin embedded prostate tissues in 8 prostate cancer samples. SOX7 was strongly methylated while WIF1, SFRP1, DKK3, and APC were partially methylated in multiple patient samples. U = unmethylated, M = methylated. **(B)** MSP analysis indicates no change in methylation status of APC, WIF1, SFRP1, SOX7, and DKK3 in DU145, ARCAPE, and PC3 cell lines after treatment with 20 μM genistein for 6 days. **(C)** Whole genome methylation profiling using the 27 K CpG Methylation Arrays was performed on ARCAPE and ARCAPM cells treated with DMSO and 20 μM genistein for 6 days. In addition, 5-aza-deoxy-cytidine was used as a positive control and PREC cells were used as a negative control. No significant changes in methylation were detected with genistein treatment. Unsupervised hierarchical clustering of 4,190 CpG loci with β-value > 0.5 is shown. **(D)** Bisulfite sequencing of 175 bp of the WIF1 CpG island across 13 CpG sites indicates high methylation in ARCAP-E and ARCAP-M cells with or without genistein treatment, and low methylation in PrEC cells. **(E)** Anti-acetyl histone H3-Lysine9 chromatin immunoprecipitation (acetyl-H3K9 ChIP) of ARCAP-E cells treated with DMSO control or genistein shows marked increases in acetyl-H3K9 following genistein treatment. **(F)** Immunoblot of HAT1 protein shows increased HAT1 protein in ARCaP-E and ARCaP-M after genistein treatment. **(G)** Gene expression with genistein treatment in ARCAPE, and ARCAPM cells. Gene expression of indicated Wnt inhibitory genes after treatment with genistein was determined using QPCR. The data are presented as fold change relative to DMSO control (mean ± SD, triplicate samples from five independent experiments). Significant p-values (p < 0.05) were computed using the student’s *t*-test with a two-tailed distribution and are indicated with an asterisk (*).

Tumor suppressor genes are often hypermethylated in prostate cancer patient tissue samples compared to normal tissues, and this methylation can correlate with prognosis [34,35]. To determine if Wnt Inhibitory genes are methylated in prostate cancer patient samples, we performed methylation specific PCR (MSP) on eight prostate cancer patient samples. We observed that SOX7 was highly methylated, whereas WIF1, SFRP1, DKK3, and APC were partially methylated in each of these samples (Figure 1A).

### **Genistein treatment does not induce demethylation of wnt-inhibitory genes but does induce expression and H3K9 acetylation in prostate cancer cells**

To test our hypothesis that genistein could demethylate Wnt inhibitory genes, APC, SOX7, SFRP1, DKK3, and WIF1 were tested for demethylation by MSP in DU145, PC-3, and ARCaP-E cells following treatment for 6 days with 20 μM genistein or with 5-aza as a positive control. Although some studies have used concentrations as high as 50 μM [20], other previously published studies have used 20 μM [17], and analysis of genistein concentrations in the prostates of patients supplemented with 82 mg/day determined that the median concentration of genistein in the prostate was only 2.3 μM, suggesting that achieving 50 μM genistein in patients is likely not attainable [23]. 5-aza treated cells demonstrated significant demethylation of SOX7 in PC3 and DU145 cells (Additional File 2: Figure S1), confirming the sensitivity of the MSP assay. Although previous reports indicated that genistein has the potential to demethylate CpG dinucleotides [17,20], we observed no demethylation of APC, SOX7, WIF1, or SFRP1 in DU145, PC3, or ARCaP-E cells when treated with 20 μM genistein (Figure 1B and Additional File 2: Figure S1). In addition, we performed whole genome methylation profiling of ARCaP-E and ARCaP-M cells treated with DMSO, 1 μM 5-aza, or 20 μM genistein for 6 days. Consistent with the MSP data, we observed no significant changes in methylation following genistein treatment across over 14,000 genes tested by this platform (Figure 1C and Additional File 2: Figure S1). In contrast, ARCaP-E and ARCaP-M cells treated with 1 μM 5-aza, exhibited a substantial change in methylation. Furthermore, bisulfite sequencing was performed on 13 CpGs over 175 basepairs of the WIF1 CpG island to obtain over 1000 reads per genomic DNA sample by next generation sequencing methods on an Ion Torrent Personal Genome Machine. Analysis of these 13 CpGs in the WIF1 CpG island in ARCAP-E, ARCAP-M, and PrEC cells indicated no change in CpG methylation upon genistein treatment (Figure 1D). Thus, we conclude that treatment with 20 μM genistein for 6 days does not induce CpG demethylation in prostate cancer cells.

It has also been previously reported that genistein can affect histone acetylation [18,36]. Consequently, we tested the effect of genistein by ChIP assay and observed that it did produce substantial changes in H3K9 acetylation in the promoters of Wnt inhibitory genes. ARCaP-E cells treated with genistein for 6 days at 20 μM demonstrated an increase in acetylation in SOX7, APC, DKK3, WIF1, SFRP1, and SFRP2 (Figure 1E). Additionally, there was an increase in the histone acetyltransferase 1 (HAT1) protein when treated with genistein (Figure 1F).

To determine if genistein treatment would induce gene expression of Wnt inhibitory genes, we performed quantitative real time PCR (QPCR) to determine if there was an increase in the mRNA levels of SOX7, SFRP2, SFRP1, APC, and DKK3. We did not observe any significant increases in ARCAP-E cells following genistein treatment, although there was a small but significant increase in SOX7 and SFRP1 expression in ARCAP-M cells (Figure 1G).

### **Genistein treatment reduces proliferation and induces apoptosis in prostate cancer cells alone or in combination with vorinostat**

**Figure 2:**
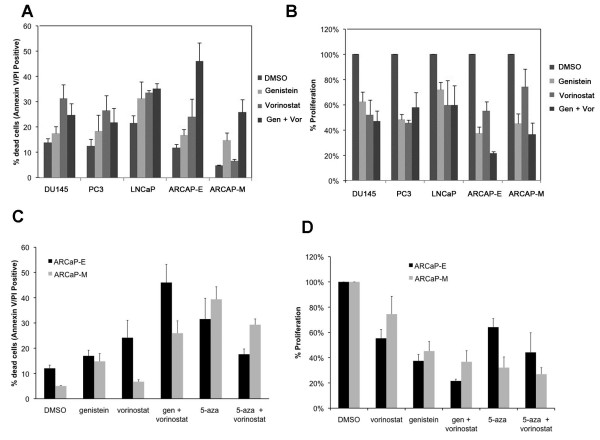
**Genistein synergizes with vorinostat to induce apoptosis in prostate cancer cells. (A)** Genistein treatment induces apoptosis in prostate cancer cells. Survival of androgen-independent PC-3 and DU145, androgen-dependent LNCaP, and androgen-repressed ARCaP-E and ARCaP-M cells in response to genistein, vorinostat, and combination treatment was measured using Annexin V/PI staining. Cells were treated with 20 μM genistein or DMSO for 6 days, 1 μM vorinostat for 2 days, and combination. Apoptosis was quantitated using Annexin V/PI staining. The data were quantitated showing total cell death. Data are presented as mean ± SE of triplicate experiments. **(B)** Proliferation of DU145, PC3, LNCAP, ARCaP-E, and ARCaP-M cells treated with genistein, vorinostat, and combination. Cells were treated with single agents vorinostat (1 μM), genistein (20 μM), or combination. Mean % proliferation ± SE of triplicate experiments are shown. Growth inhibition was measured using a hemocytometer following trypan blue staining. **(C)** Cell death of ARCaP-E and ARCaP-M cell treated with genistein**,** 5-deoxyazacytidine (5-aza), and vorinostat. Cells were treated with single agents, 1 μM vorinostat for 48 hrs, or in combination with genistein or 5-aza for 6 days. Total mean cell death ± SE of triplicate experiments are shown. **(D)** Cells were treated as in **(C)** and cell proliferation was assessed**.** Mean % proliferation ± SE of triplicate experiments are shown.

To determine genistein’s potential as a therapeutic agent in the treatment of prostate cancer, prostate cancer cell lines PC3, DU145, ARCaP-E, ARCaP-M and LNCaP were treated with 20 μM genistein for a total of six days, 1 μM vorinostat for 2 days, and a combination of genistein and vorinostat (Figure 2A). Since genistein treatment increased histone acetylation, we hypothesized that it might cooperate with histone deacetylase (HDAC) inhibitors to induce apoptosis. Genistein exhibited only a minor (5–10 %) effect of increased cell death on these cells based on Annexin V/PI staining (Figure 2A). There was an approximate increase of 8 % cell death in DU145 cells, 5 % cell death in ARCaP-E cells, 10 % cell death in LNCaP cells, and 8 % cell death in PC3 cells when compared to untreated DMSO cohorts. Nevertheless, we confirmed previous studies [37,38] indicating that genistein was quite effective in inhibiting cell proliferation (Figure 2B). In addition, there was an increase in cell death of all prostate models when treated with vorinostat and combination genistein and vorinostat with the largest affect being in the ARCaP-E cell line model.

To compare the effectiveness of genistein and 5-aza, ARCaP-E and ARCaP-M cells were treated with either 20 μM genistein or 1 μM 5-aza, in combination with the HDAC inhibitor vorinostat for 48 hrs. Combination treatment of vorinostat and genistein showed that there was a more than additive effect on cell death in both ARCaP cell lines (Figure 2C). Also, these data suggest that prostate cancer cells that undergo EMT may become less sensitive to vorinostat treatment. Although 5-aza, as a single agent, increased cell death more than genistein as a single, when 5-aza was used in combination with vorinostat, there was no substantial increase in cell death. Interestingly, in ARCaP-E cells, the combined 5-aza and vorinostat actually produced less cell death than both agents used separately. In addition, genistein combined with vorinostat was much more effective than 5-aza combined with vorinostat in inducing cell death (Figure 2C). This is potentially of significant clinical relevance because genistein has no known toxicities.

In addition to genistein’s effects on cell death, we also measured genistein’s effects on cellular proliferation. In ARCaP-E cells, we observed that genistein was more effective in inhibiting cell growth than either vorinostat or 5-aza (Figure 2D). There was greater than 60 % and 50 % inhibition of growth in ARCaP-E and ARCaP-M cell lines, respectively, following genistein treatment. ARCaP-E cells treated with 5-aza showed only a 35 % reduction in proliferation, whereas ARCaP-M cells had a greater than 50 % inhibition of growth with 5-aza alone (Figure 2D). Importantly, the combination of genistein and vorinostat decreased proliferation by 80 % in ARCaP-E and greater than 60 % in ARCaP-M cells. These data show that genistein is more effective than 5-aza in inhibiting cell proliferation in ARCAP-E cells, and that combined genistein and vorinostat has a profound inhibition on cellular proliferation.

### **DNA damage checkpoint and apoptosis networks identified by whole genome expression profiling of cells treated with genistein and vorinostat**

**Figure 3:**
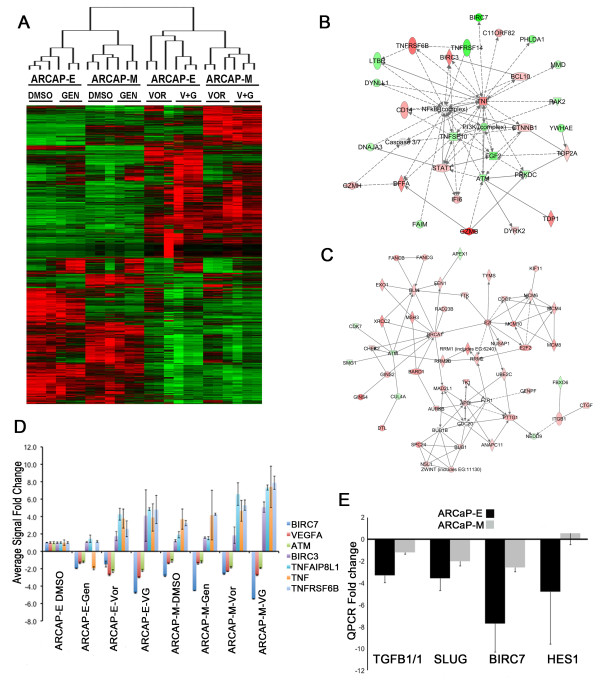
**Whole genome expression profiling of prostate cancer cells treated with genistein, vorinostat, or the combination. (A)** Hierarchical clustering of 1077 probes strongly affected by treatment with genistein and vorinostat. **(B)** Ingenuity Pathway Analysis (IPA) Network of genes annotated for function in Apoptosis. Red indicates increased expression after treatment, and green indicates reduced expression. Solid lines represent direct interactions and dashed lines represent indirect interactions. **(C)** IPA Network of genes annotated for function in DNA Repair and Cell Cycle. **(D)** QPCR data from a subset of genes with functions in apoptosis pathways normalized to ARCAP-E control cells. **(E)** QPCR confirmation of down regulation of TGFB1I1/ARA55, SLUG, BIRC7/Livin, and HES1 following genistein treatment. Data are presented as mean ± SE of triplicate experiments.

To determine the genome-wide effects of genistein, vorinostat, and combined treatment on gene expression, we conducted whole genome expression profiling of ARCaP-E and ARCaP-M cells using Illumina HT-12 v3 Expression BeadChips. Genistein treatment had a larger effect on ARCaP-E cells (291 genes induced and 144 genes repressed) than on ARCaP-M cells (31 genes induced and 33 repressed). Vorinostat impacted more genes than genistein in both ARCaP-E cells (820 genes induced and 1046 genes repressed) and ARCaP-M cells (1296 genes induced and 883 genes repressed). As expected, the largest changes in gene expression were observed by combined treatment with genistein and vorinostat for ARCaP-E cells (1978 genes induced and 1758 genes repressed) and ARCaP-M cells (1503 genes induced and 1161 genes repressed) (Figure 3A). Gene ontology enrichment analysis using the DAVID knowledgebase [39] and Ingenuity Pathway Analysis (IPA) [40], demonstrated that the affected genes were highly enriched in genes involved in DNA damage, cell cycle arrest, and apoptosis (Tables 1 and 2). Interestingly, IPA analysis of genes affected by combined genistein and vorinostat treatment identified a gene network with the pro-apoptotic Tumor Necrosis Factor alpha (TNFα) as a major hub (Figure 3B), including the pro-survival gene BIRC7 (or Livin) which was downregulated 4.8-fold. In addition, genes involved in the G2/M cell cycle and response to DNA damage was also identified (Figure 3C-D). For example, four members of the minichromosome maintenance complex (MCM) essential for DNA replication are strongly upregulated, as are BRCA1, BARD1, RAD23B, and XRCC2. QPCR analysis following genistein treatment confirmed reduced levels of BIRC7, as well as SLUG, HES1, and TGFB1I1 (or ARA55), and several genes associated with apoptosis (Figure 3E). These data indicate that genistein affects cell survival and proliferation via multiple mechanisms including the TNFα-NFκB and ATM-CHEK2-BRCA1 pathways. Additionally, genes involved in chromatin modifications and histone acetylations such as HAT1 were also impacted by genistein treatment (Table 1 and Additional File 3: Figure S2).

**Table 1:** Fold changes in ARCAP-E cells (combined vorinostat/genistein vs. DMSO) of critical genes involved in apoptosis, DNA damage checkpoint, and chromatin structure and remodeling

***Symbol***	***Synonym***	***Fold Change***	***Network***	***Symbol***	***Synonym***	***Fold Change***	***Network***
GZMB	Granzyme B	6.9	Apoptosis	CTNNB1	β-catenin	1.7	Apoptosis
TNFRSF6B	Decoy Receptor 3	4.8	Apoptosis	MTA2	---	1.7	Chromatin
BIRC3	c-IAP2	4.1	Apoptosis	CHEK2	RAD53	1.6	DNA Checkpoint
TNF	TNFα	3.9	Apoptosis	BUB1	---	1.6	DNA Checkpoint
DFFA	ICAD	3.8	Apoptosis	TGFB1I1	ARA55	−1.8	Apoptosis
BARD1	BRCA1 Associated	3.0	DNA Checkpoint	TNFSF10	TRAIL	−1.9	Apoptosis
MAD2L1	---	2.4	DNA Checkpoint	CREBBP	CBP	−2.1	Chromatin
HAT1	Histone Acetyltrans-ferase 1	2.4	Chromatin	SIRT1	SIR2L1	−2.1	Chromatin
GZMH	Granyme H	2.3	Apoptosis	ATM	TEL1	−2.2	DNA Checkpoint
AURKB	Aurora Kinase B	2.2	DNA Checkpoint	TNFRSF14	LIGHTR	−3.7	Apoptosis
BCL10	---	2.0	Apoptosis	BIRC7	Livin	−4.8	Apoptosis
BRCA1	---	1.9	DNA Checkpoint				

**Table 2:** Gene ontology analysis of genes affected by combined genistein and vorinostat treatment using DAVID knowledgebase (columns 1–4) and ingenuity knowledgebase (columns 5–7)

***GO Term***	***Biological Process***	***Count***	***p-value***	***IPA Biological Function***	***Count***	***p-value***
GO:0006281	DNA repair	45	1.03E-13	DNA Replication, Recombination, and Repair	39	7.18E-12
GO:0008219	Cell Death	48	2.73E-03	Cell Death	122	1.95E-10
GO:0022403	Cell Cycle	57	1.63E-14	Cell Cycle	105	2.70E-09
GO:0006915	Apoptosis	43	1.37E-03	Apoptosis	149	1.13E-07
GO:0000075	Cell Cycle Checkpoint	17	1.45E-06	DNA checkpoint control	13	1.90E-06
GO:0006325	Chromatin Organization	28	7.01E-03	Binding Of Chromatin	5	1.69E-03

## Discussion

Genistein is a pleiotropic compound with many clinically attractive properties. Previous studies have suggested that some of the mechanisms of action of genistein includes inhibition of tyrosine kinases and NFκB, DNA CpG demethylation, and other mechanisms [3,5,6,18]. Initially, we hypothesized that genistein would demethylate Wnt inhibitory genes and induce their expression, possibly inhibiting the Wnt pathway. Although previous studies showed that genistein demethylates CpG dinucleotides at 50 μM [20], our experiments showed no effect at 20 μM. Moreover, analysis of genistein concentrations in the prostates of patients supplemented with 82 mg/day determined that the median concentration of genistein in the prostate was only 2.3 μM [23]. Despite the fact that genistein has a low toxicity and is well tolerated at high concentrations in individuals, high concentrations of genistein that are capable of demethylating DNA in a physiological setting may not be clinically feasible. Nevertheless, genistein may still prove to be useful clinically as a chemopreventative or as a therapeutic agent in combination therapy with drugs such as vorinostat, since combining genistein with vorinostat demonstrated a more than additive effect on inducing cell death (Figure 2A). Genistein was also tested in combination with a Notch inhibitor (DAPT), Wnt inhibitor (ICG-001), and an AKT inhibitor (LY-294002). However, those treatments in combination did not prove to be as effective in inducing cell death in prostate cancer cells as combination genistein and vorinostat (Additional File 4: Figure S3).

Previous studies have reported that genistein reduces cell growth and induces apoptosis in a number of cancer cells [8]. Our data suggest that this may be as a result of increase in genes that affect the G2/M checkpoint such as BRCA1, BARD1, BUB1, AURKB, CHEK2, and MAD2L1 as well as genes involved in apoptosis such as GZMB, DFFA, TNF, BIRC3, BCL10, and BIRC7/Livin (Tables 1 and 2). These data provide evidence for potential mechanisms to explain earlier studies showing that genistein sensitizes prostate cells to treatment with docetaxel and selenium [41,42].

Our data from whole genome expression analysis and real time PCR validation suggest that the mechanism of action of cell death from genistein may be due to down regulation of anti-apoptotic genes such as BIRC7/Livin, TGFB1I1/ARA55, HES1, and SLUG that are involved in the TNF-NFκB and the androgen pathway (Figure 3). SLUG (a.k.a. SNAI2) plays a critical role in mediating the EMT [43] along with SNAIL [44], the founding member of this family of transcription factors. HES1 is a downstream target and mediator of the Notch signaling pathway [45], suggesting that genistein treatment may inhibit Notch pathway activity. TGFB1I1/ARA55 is induced by TGBβ signals and acts as a co-activator for the androgen receptor [46], suggesting that both TGBβ signaling and androgen signaling may be inhibited by genistein. We observed that ARCaP-E cells are more sensitive to genistein and vorinostat treatment than ARCaP-M cells (Figure 2C), and we also determined that there was a greater decrease in BIRC7/Livin, TGFB1I1/ARA55, HES1, and SLUG in ARCaP-E cells compared to ARCaP-M cells (Figure 3E), suggesting possible mechanisms for chemotherapeutic resistance in mesenchymal cells compared to epithelial cells.

## Conclusion

The effects of genistein treatment on epigenetics and gene expression are likely due primarily to changes in histone acetylation rather than CpG methylation. Similar to previous reports [19,20] we observed an increase of HAT1 upon genistein treatment in our array data (Table 1) and increase protein levels of HAT1 (Figure 1E). This change in HAT1 expression may provide a mechanism for increased H3K9 acetylation and explain why Wnt inhibitory genes such as SOX7 were slightly induced when treated with genistein despite a lack of change in CpG methylation. We observed changes in a large number of genes and pathways that were affected with combinatorial effects of genistein and vorinostat including the TNF-NFκB pathway and G2/M cell cycle arrest in response to DNA damage and repair (Figure 3C and Tables 1 and 2).

In conclusion, we have shown that genistein can cooperate with vorinostat to induce apoptosis, however, future studies are needed to validate this combination in a clinical setting.

## Competing interests

The authors declare that they have no competing interests.

## Authors’ contributions

The authors contributions are the following: CJP contributed with experimental design, collection and analysis of data, interpretation of findings, and writing of the manuscript. CKG contributed with experimental design, collection and analysis of data, and editing of the manuscript. YL contributed with collection and analysis of the data and editing of the manuscript. DJC performed Ion Torrent Data sequence analysis. OK contributed with experimental design, provided prostate patient samples, and editing of the manuscript. CSM contributed with experimental design, analysis of data, interpretation of findings, and editing of the manuscript. All authors read and approved the final manuscript.

## Pre-publication history

The pre-publication history for this paper can be accessed here:

http://www.biomedcentral.com/1471-2407/12/145/prepub

## Supplementary Material

Additional file 1 **Table S1.** Clinical characteristics of patient samples. **Table S2.** Primer sequences for methylated and unmethylated genes. **Table S3.** Primer sequences for ChIP assay. **Table S4.** Primer sequences for Real time RT PCR. Click here for file

Additional file 2 **Figure S1. (A)** DU145 and PC3 cells were treated with increased doses 5-deoxy-azacytidine or genistein, and methylation of SOX7 was assessed by MSP. Although there is an apparent slight decrease in methylated SOX7 in PC3 cells, there is no corresponding increase in unmethylated SOX7 in these cells. Moreover, there is no change in SOX7 methylation in DU145 cells. **(B)** Histogram of beta-values representing the global level of CpG methylation in the ARCaP-E cell model when treated with DMSO, genistein, or 5-aza. Click here for file

Additional file 3 **Figure S2.** Network of genes annotated for function in chromatin structure and remodeling. Click here for file

Additional file 4 **Figure S3. (A)** Combination cell death with genistein for 6 days in combination with 5 μM vorinostat, 10 μM DAPT, 10 μM ICG-001, and 20 μM LY-294002 for 48 hrs in DU145 cells. **(B)** Combination cell death with genistein for 6 days in combination with 5 μM vorinostat, 10 μM DAPT, 10 μM ICG-001, and 20 μM LY-294002 for 48 hrs in ARCaPE cells. Click here for file
