# Supplementary material for: Genistein cooperates with the histone deacetylase inhibitor vorinostat to induce cell death in prostate cancer cells
Source: BMC Cancer. 2012 Apr 11;12:145. doi: 10.1186/1471-2407-12-145 (PMC3472186; doi:10.1186/1471-2407-12-145)
Supplement: Additional file 1 — Table S1. Clinical characteristics of patient samples. Table S2. Primer sequences for methylated and unmethylated genes. Table S3. Primer sequences for ChIP assay. Table S4. Primer sequences for Real time RT PCR. [file 1471-2407-12-145-S1.docx]

| **Patient ID** | **Gleason score** | **Time (Months)** | **Recurrence** | **Age** | **Stage** | **PSA** |
| --- | --- | --- | --- | --- | --- | --- |
| PT30 | 7 | 160.1 | Non-Recurrence | 54 | 2b | 14.1 |
| PT59 | 7 | 56.1 | Non-Recurrence | 54 | 3a | 5.6 |
| PT60 | 7 | 3 | Non-Recurrence | 64 | 2a | 11 |
| PT85 | 7 | 164.1 | Non-Recurrence | 67 | 2a | 8.3 |
| PT89 | 7 | 85.1 | Non-Recurrence | 50 | 2b | 5.3 |
| PT110 | 7 | 10 | Recurrence | 62 | 4a | 16 |
| PT119 | 7 | 24 | Recurrence | 68 | 2a | 1.26 |
| PT83 | 7 | 54 | Recurrence | 63 | 2 | 15.2 |

**Supplemental Table S1:** Clinical characteristics of patient samples.

**Supplemental Table S2:** Primer sequences for methylated and unmethylated genes

| **Primer name** | **Sequence** | **Product size (bp)** | **Annealing Temp (°C)** |
| --- | --- | --- | --- |
| APC Left Methylated Primer | TATTGCGGAGTGCGGGTC | 98 | 55 |
| APC Right Methylated Primer | TCGACGAACTCCCGACGA |  |  |
| APC Left Unmethylated Primer | GTGTTTTATTGTGGAGTGTGGGTT | 108 | 60 |
| APC Right Unmethylated Primer | CCAATCAACAAACTCCCAACAA |  |  |
|  |  |  |  |
| DKK3 Left Methylated Primer | GTATTCGAGGGAGTTCGTAAGAC | 164 | 53.4 |
| DKK3 Right Methylated Primer | TAAAACGTAAAACGTAAAATAACCG |  |  |
| DKK3 Left Unmethylated Primer | GGTATTTGAGGGAGTTTGTAAGATG | 165 | 51.4 |
| DKK3 Right Unmethylated Primer | TAAAACATAAAACATAAAATAACCA |  |  |
|  |  |  |  |
| SOX7 Left Methylated Primer | TGTCGTAGAGACGTAAGGTAGGC | 226 | 54 |
| SOX7 Right Methylated Primer | TACGATAACCGAAACCCGAT |  |  |
| SOX7 Left Unmethylated Primer | GTAGGTGTTGTAGAGATGTAAGGTAGGT | 240 | 54 |
| SOX7 Right Unmethylated Primer | AACTAAAACTACAATAACCAAAACCCA |  |  |
|  |  |  |  |
| WIF1 Left Methylated Primer | TTTTAGAGTTAGAGCGCGGC | 105 | 54 |
| WIF1 Right Methylated Primer | CAAATTACGTAAATACGACACGAA |  |  |
| WIF1 Left Unmethylated Primer | GATGTTTTAGAGTTAGAGTGTGGTGG | 110 | 54 |
| WIF1 Right Unmethylated Primer | CCAAATTACATAAATACAACACAAA |  |  |
|  |  |  |  |
| SFRP1 Left Methylated Primer | GGTCGAGTTTACGGTTAGAAGC | 172 | 54 |
| SFRP1 Right Methylated Primer | TAAAAATTTACGACAAAACGCG |  |  |
| SFRP1 Left Unmethylated Primer | GTTGGTTGAGTTTATGGTTAGAAGTG | 178 | 54 |
| SFRP1 Right Unmethylated Primer | CCCTAAAAATTTACAACAAAACACAC |  |  |
|  |  |  |  |
| SFRP2 Left Methylated Primer | TTTTAGATGTCGTCGTTCGTTC | 153 | 52.6 |
| SFRP2 Right Methylated Primer | AATACCTAATCGCGAAACCG |  |  |
| SFRP2 Left Unmethylated Primer | TTTTAGATGTTGTTGTTTGTTTGT | 155 | 51.4 |
| SFRP2 Right Unmethylated Primer | CCAATACCTAATCACAAAACCACT |  |  |

**Supplemental Table S3:** Primer sequences for ChIP assay

| **Primer name** | **Sequence** |
| --- | --- |
| DKK3 chip Forward | CAGGAACCTGGAGCTGAGTC |
| DKK3 chip Reverse | GACCGCATGGTAGAAGGTGT |
|  |  |
| SOX7 chip Forward | TCACCGGTAAAATGGGGATA |
| SOX7 chip Reverse | CTGAAATTCCTCCGTTTGGA |
|  |  |
| WIF1 chip Forward | TTGCATCCAAAGCTTCACAG |
| WIF1 chip Reverse | CCACTCGCTCCGAGTTAAAG |
|  |  |
| SFRP1 chip Forward | TCTCAGCCTCCCAGAGTGTT |
| SFRP1 chip Reverse | TCTTCCTGGCTGCTTACGAT |
|  |  |
| APC chip Forward | GCTCATACTGGAGCGGTAGC |
| APC chip Reverse | CCCTGTGGACACCTTGACTT |
|  |  |
| SFRP2 chip Forward | CGAGAAGAACGATGCGTGTA |
| SFRP2 chip Reverse | GTTGGAGACACCCGACAAGT |

**Supplemental Table S4:** Primer sequences for Real time RT PCR

| **Primer name** | **Sequence** |
| --- | --- |
| DKK3 Forward | GATGCCCTTGTGCCAGT |
| DKK3 Reverse | TGCCAACTTCATACTCATCGG |
|  |  |
| SOX7 Forward | CACAACGCCGAGCTCAG |
| SOX7 Reverse | GGCCGGTACTTGTAGTTGG |
|  |  |
| APC Forward | GCTCTATGAAAGGCTGCATGAG |
| APC Reverse | TCACACTTCCAACTTCTCGC |
|  |  |
| WIF1 Forward | AGAAGGTTGGCATGGAAGAC |
| WIF1 Reverse | CAGATGTAATTGGATTCAGGTGG |
|  |  |
| SFRP1 Forward | AAGTGTGACAAGTTCCCCG |
| SFRP1 Reverse | TGGCCTCAGATTTCAACTCG |
|  |  |
| SFRP2 Forward | TTGAGTGCGACCGTTTCC |
| SFRP2 Reverse | AAGCGTTTCCATTATGTCGTTG |
|  |  |
| BIRC7 Forward | TGGGCATATTCTGAGATTGGC |
| BIRC7 Reverse | ACTTGGCACTGTCTTTAGGTC |
|  |  |
| TGFBI/1 (ARA55) Forward | CTCTGTGAGCTAGATCGGTTG |
| TGFBI/1 (ARA55) Reverse | GGAGGCTGGGTCTTTTCTTATC |
|  |  |
| SLUG (SNAI2) Forward | ACTGCTCCAAAACCTTCTCC |
| SLUG (SNAI2) Reverse | TGTCATTTGGCTTCGGAGTG |
|  |  |
| HES-1 Forward | AGGCGGCTA AGGTGT TTG |
| HES-1 Reverse | GAAGAGAGGTGGGTTGGG |
