# Supplementary figures and images for: Genistein cooperates with the histone deacetylase inhibitor vorinostat to induce cell death in prostate cancer cells
Source: BMC Cancer. 2012 Apr 11;12:145. doi: 10.1186/1471-2407-12-145 (PMC3472186; doi:10.1186/1471-2407-12-145)

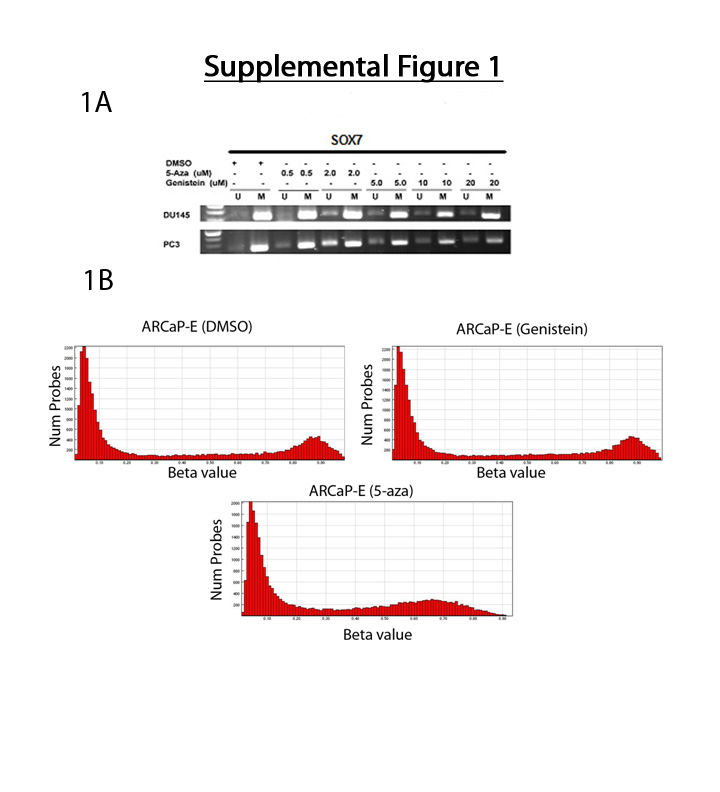

Supplement: Additional file 2 — Figure S1. (A) DU145 and PC3 cells were treated with increased doses 5-deoxy-azacytidine or genistein, and methylation of SOX7 was assessed by MSP. Although there is an apparent slight decrease in methylated SOX7 in PC3 cells, there is no corresponding increase in unmethylated SOX7 in these cells. Moreover, there is no change in SOX7 methylation in DU145 cells. (B) Histogram of beta-values representing the global level of CpG methylation in the ARCaP-E cell model when treated with DMSO, genistein, or 5-aza. [file 1471-2407-12-145-S2.jpeg]

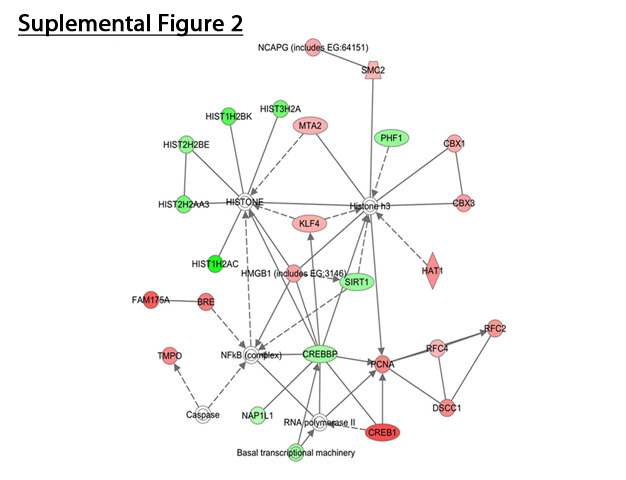

Supplement: Additional file 3 — Figure S2. Network of genes annotated for function in chromatin structure and remodeling. [file 1471-2407-12-145-S3.jpeg]

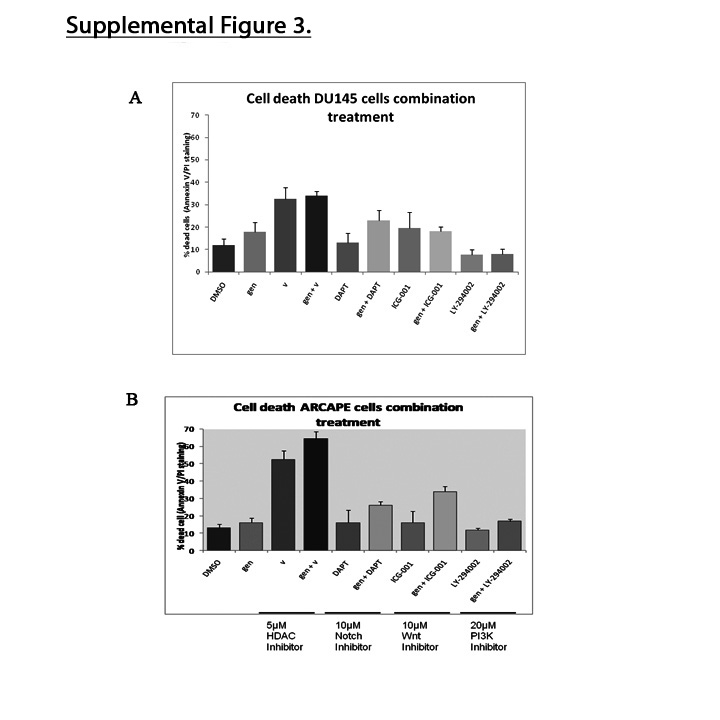

Supplement: Additional file 4 — Figure S3. (A) Combination cell death with genistein for 6 days in combination with 5 μM vorinostat, 10 μM DAPT, 10 μM ICG-001, and 20 μM LY-294002 for 48 hrs in DU145 cells. (B) Combination cell death with genistein for 6 days in combination with 5 μM vorinostat, 10 μM DAPT, 10 μM ICG-001, and 20 μM LY-294002 for 48 hrs in ARCaPE cells. [file 1471-2407-12-145-S4.jpeg]
